# Home detection of freezing of gait using support vector machines through a single waist-worn triaxial accelerometer

**DOI:** 10.1371/journal.pone.0171764

**Published:** 2017-02-15

**Authors:** Daniel Rodríguez-Martín, Albert Samà, Carlos Pérez-López, Andreu Català, Joan M. Moreno Arostegui, Joan Cabestany, Àngels Bayés, Sheila Alcaine, Berta Mestre, Anna Prats, M. Cruz Crespo, Timothy J. Counihan, Patrick Browne, Leo R. Quinlan, Gearóid ÓLaighin, Dean Sweeney, Hadas Lewy, Joseph Azuri, Gabriel Vainstein, Roberta Annicchiarico, Alberto Costa, Alejandro Rodríguez-Molinero

**Affiliations:** 1 Universitat Politècnica de Catalunya – BarcelonaTech (UPC), Technical Research Centre for Dependency Care and Autonomous Living (CETPD), Vilanova i la Geltrú, Spain; 2 Sense4Care, Barcelona, Spain; 3 Unidad de Parkinson y trastornos del movimiento (UParkinson), Barcelona, Spain; 4 School of Medicine, National University of Ireland Galway (NUIG), Galway, Ireland; 5 Electrical & Electronic Engineering Department, National University of Ireland Galway (NUIG), Galway, Ireland; 6 Maccabi Healthcare Services, Tel Aviv, Israel; 7 Sackler Faculty of Medicine, Tel Aviv University, Tel Aviv, Israel; 8 IRCCS Fondazione Santa Lucia, Rome, Italy; Universitat Rovira i Virgili, SPAIN

## Abstract

Among Parkinson’s disease (PD) symptoms, freezing of gait (FoG) is one of the most debilitating. To assess FoG, current clinical practice mostly employs repeated evaluations over weeks and months based on questionnaires, which may not accurately map the severity of this symptom. The use of a non-invasive system to monitor the activities of daily living (ADL) and the PD symptoms experienced by patients throughout the day could provide a more accurate and objective evaluation of FoG in order to better understand the evolution of the disease and allow for a more informed decision-making process in making adjustments to the patient’s treatment plan. This paper presents a new algorithm to detect FoG with a machine learning approach based on Support Vector Machines (SVM) and a single tri-axial accelerometer worn at the waist. The method is evaluated through the acceleration signals in an outpatient setting gathered from 21 PD patients at their home and evaluated under two different conditions: first, a generic model is tested by using a leave-one-out approach and, second, a personalised model that also uses part of the dataset from each patient. Results show a significant improvement in the accuracy of the personalised model compared to the generic model, showing enhancement in the specificity and sensitivity geometric mean (GM) of 7.2%. Furthermore, the SVM approach adopted has been compared to the most comprehensive FoG detection method currently in use (referred to as MBFA in this paper). Results of our novel generic method provide an enhancement of 11.2% in the GM compared to the MBFA generic model and, in the case of the personalised model, a 10% of improvement with respect to the MBFA personalised model. Thus, our results show that a machine learning approach can be used to monitor FoG during the daily life of PD patients and, furthermore, personalised models for FoG detection can be used to improve monitoring accuracy.

## Introduction

Parkinson’s disease (PD) is a neurodegenerative disease that principally affects the motor system. According to the Global Declaration for Parkinson’s Disease, PD affects up to 6.3 million people worldwide [1]. Among the many PD symptoms, Freezing of Gait (FoG) is one of the most incapacitating and is usually present in the more advanced phase of the disease [2,3]. FoG is commonly described by PD patients as if their feet were “glued to the floor” resulting in loss of postural balance frequently causing falls [4,5]. In addition to the motor complications arising from FoG, it can also lead to non-motor complications including social isolation, depression, and anxiety. [6,7].

The precise tracking of the occurrence of FoG is challenging for clinicians. The acquisition of such information can greatly help in optimising therapies including pharmacotherapy, and physiotherapy, that are known to be beneficial for reducing FoG episodes [4]. Furthermore, by acquiring accurate information on FoG frequency and severity in conjunction with the full repertoire of other motor and non-motor symptomatology, this would provide to the neurologists a more accurate overall view of the disease status and evolution over time. Additionally, the online detection of FoG opens up the possibility of incorporating actuation methodologies such as rhythmic auditory cues that might shorten or prevent FoG episodes [8,9]. These rhythmic auditory cues are hereafter referred to as *cueing*.

In clinical practice, there are two methods employed to evaluate the presence of FoG. The first is based on specific gait tests, for example the Timed Up & Go test performed through a narrow space, using specific circuits with several turns [10–12] or by assessing patients while performing rapid and short steps [13]. The second method is based on answers given by patients and caregivers about the frequency and severity of FoG episodes collected in specific FoG questionnaires [14–16]. These methods have major drawbacks due to both the clinical setting not reflecting real life settings as well as the timing of the evaluation not replicating the recognised variability in FOG frequency. FoG episodes often occur in the patient’s own home and their frequency is reduced significantly in controlled environments such as the laboratory setting [17]. Self-assessment of FoG, has shown to be unreliable since patients and caregivers may not perceive the episodes and, furthermore, because PD patients often experience memory loss, inattention or dementia, leading to imprecise recall [18].

Therefore, many attempts have been made to obtain an objective and precise method for monitoring FoG in patients during their normal activities of daily living (ADL). In this context, many different types of sensors have been employed. The most widespread method to objectively detect FoG is based on the analysis of data emanating from wearable motion sensors, as they are unobtrusive, portable with low power consumption, providing currently the most suitable way to evaluate FoG episodes in patients’ own homes.

Most methods to detect FoG based on wearable sensors have been developed and tested under controlled conditions [19–27], which is mainly due to the difficulty of obtaining a valid gold standard outside the laboratory setting. Therefore, trained algorithms are likely to generate several false positives when they are tested in real life since algorithms have not been trained for specific situations that could happen in patients daily living activities. As far as the authors are aware, only Ahlrichs et al. have generated an algorithm to detect FoG in PD patients’ own homes [28]; however, part of the evaluation was not performed with PD patients with FoG and the latency time of the algorithm was every minute, which is too slow to be effective for cueing purposes. Until now, objective FoG assessment during ADLs in the home has not been extensively examined, thus, it might not be reliable. The usability and acceptability of any new wearable device is critical to its success. Many studies have employed several sensors at specific locations (Table 1) providing promising results in some cases. However, their usage may be counterproductive when monitoring FoG at home, as they require patients to wear many sensors, reducing the usability of the monitoring system and its acceptability. Thus, the trade-off between accuracy and usability is a key factor in designing such systems. The waist is one of the most suitable placements for detecting FoG [28–31]. Furthermore, according to Mathie et al., locating a wearable system at a side of the waist is the most comfortable place, as concluded in a questionnaire performed by elderly people [32]. Additionally, in humans the waist is close to the centre of gravity better representing body movement [33] and also allowing the sensor to monitor other PD symptoms such as bradykinesia or dyskinesia [34,35].

**Table 1:** Summary of literature regarding patients and sensor systems.

*Work*	*Year*	*# Patients*			*Sensors*	*# Sensors*	*Location*
		PD-Freezers*	*PD Non-Freezers*	*Control*			
Nieuwboer et al. [39]	2001	17(14)	-	-	Camera, Force Plates, EMG	6, 1 and 6 respectively	EMG at lower limbs, the others are not wearable
Han et al.[19]	2003	2(2)	-	-	Accelerometers	2	2 x ankle
Hausdorff et al.[38]	2003	11(11)	-	-	Insole pressure	1	Not wearable
Moore et al. [20]	2008	11(7)	-	-	Accelerometers	1	Left ankle
Daphnet project [56]	2009–2011	10 (8)	-	-	Accelerometers	1	Knee
Zabaleta et al. [24]	2008	2(2)	-	-	Accelerometers	6	2 x ankle, 2 x knee, 2 x thigh
Jovanov et al.[22]	2009	1(1)	-	4	Accelerometers	1	Ankle
Maidan et al. [57]	2010	10(10)	10	15	ECG, Insoles and acc.	2 each	ECG leads at chest, insole and acc. at shoe.
Knobl et al. [40]	2010	15(15)	16	16	Pressure platform	1	Not wearable
Delval et al.[23]	2010	10(5)	10	10	Goniometer, Video system	2	2 x Knee
Cole et al. [25]	2011	10(4)	-	2	EMG and Accelerometers	1 and 3 respectively	EMG at shin. Acc. at shin, thigh and forearm
Niazmand et al. [26]	2011	6(6)	-	-	Accelerometers	5	2 x Shank, 2 x Thigh and 1 x lower abdomen
Stamatakis et al. [51]	2011	1(1)	-	1	Accelerometers	4	2 x Hallux and 2 x heel
Almeida et al. [40]	2012	10(10)	10	10	Pressure platform	1	Not wearable
Zhao et al. [46]	2012	8(6)	-	-	Accelerometers	5	2 x shank, 2 x thigh and 1 x lower abdomen
Handojoseno et al. [42]	2012	26(10)	-	-	Encephalogram	4 electrodes	Head
Mancini et al. [47]	2012	21(21)	27	21	Accelerometers	3	1 x Posterior trunk, 2 x shank
Mazilu et al. [48]	2012	10(8)	-	-	Accelerometers	3	Shank, tight and lower back
Takac et al. [58]	2013	1(1)	-	12	Depth Sensor Camera, Accelerometers	1 each	Acc at waist
Moore et al.[27]	2013	25 (20)	-	-	Accelerometers	7	Lower back, 2xThigh, 2xShank and 2xHeel
Tripoliti et al. [52]	2013	5(5)	6	5	Accelerometers and gyroscopes	6 and 2 respectively	Acc: (2 x shank, 2 x wrist, 1 x chest, 1 x abdomen) Gyro: abdomen and chest
Mazilu et al. [49]	2013	10(8)	-	-	Accelerometers	3	Shank, tight and lower back
Rodríguez et al. [50]	2014	10(10)	10	-	Accelerometers	1	Waist
Coste et al. [53]	2014	4(4)	-	-	Accelerometer	1	Ankle
Weiss et al. [30]	2015	28(28)	44	-	Accelerometer	1	Lower Back
Tay et al. [54]	2015	8(5)	-	-	Gyroscope	2	2 x Ankle
Zach et al. [31]	2015	23(23)	-	-	Accelerometers	1	Waist
Mazilu et al. [43]	2015	18(11)	-	-	ECG and Skin Conductance	1 each	ECG at chest, SC in 2 fingers
Ahlrichs et al. [28]	2015	8(8)	12	-	Accelerometers	1	Waist
Maidan et al. [59]	2015	11 (11)	-	11	near Infrared Spectroscopy	1	Head

* In brackets the total of PD patients who suffered FoG episodes during the tests

This paper presents a study performed within the ‘Freezing in Parkinson’s Disease: Improving Quality of Life with an Automatic Control System’ (MASPARK) project [36] for detecting FoG by locating a single wearable sensor at the waist. More specifically, this paper presents a new machine learning based algorithm to detect FoG episodes validated by clinical experts, in PD patients’ own home [37]. Furthermore, and with the aim of evaluating the algorithm in real life, the proposed algorithm is also designed with the purpose of being implemented in a microcontroller, thus, some issues in the computational resources used by the method such as windowing and the classification cost have also been taken into account. The approach presented here is compared to the most extended algorithm used to detect FoG (hereafter referred to as the *Moore-Bächlin FoG Algorithm*, MBFA) [21]. The validation methodology employed for both approaches consists of a Leave-One-Patient-Out. Furthermore, this leave one out analysis is compared to a user-dependent algorithm, in which an individualised model classifier is evaluated. Results on 21 PD patients show that the generic proposed approach outperforms the current most employed FoG detection method by an average 11%, with a sensitivity of 74.7% (proportion of actual positives that are correctly identified as positives) and specificity of 79% (proportion of actual negatives correctly identified as negatives) compared to a sensitivity of 81.6% and specificity of 52.6%. In addition, personalisation of the detection model achieves an improvement in the symptom detection of around 10% on average (80.1 on sensitivity, 88.1% on specificity against an 89.1% on sensitivity and 61.5% on specificity). Thus, a machine learning approach can be used to monitor FoG during the daily life of PD patients and, furthermore, personalised models for FoG detection can be used to improve the monitoring accuracy.

The paper is organised as follows: first a background with the most relevant FoG detection systems and algorithms is introduced, then the proposed methodologies, the experiments performed and the evaluation of the algorithms are presented, and finally, the results, discussion and conclusions are provided.

## Related work

FoG has been analysed with a wide variety of systems and sensors. Some of these reports have employed systems to capture FoG episodes that are not transferable to normal daily life of patients since they can only be used in a laboratory setting. Some examples are pressure platforms [38–41], which are non-portable systems, electroencephalogram (EEG) [42], electromyography (EMG) [25] or skin conductance [43], which involves the placement of electrodes on the skin in addition to a datalogger system to capture these data. Other obtrusive system examples are knee-joint goniometers [23] or cameras and video systems, which also have a low acceptability by users in a non-laboratory setting [23,39,44]. Thus, given that PD monitoring should be ambulatory and should last several hours in order to provide useful clinical information [34,45], most works have employed non-invasive systems such as wearable systems based in microelectromechanical systems (MEMS).

In 2003, Han et al. used MEMS based inertial systems, i.e. accelerometers, to explore features to analyse FoG episodes. They found that the frequency response in 2 patients wearing accelerometers at the ankle was around 6–8 Hz [19]. In 2008, Moore et al. proposed a methodology for detecting FoG with an accelerometer at the ankle where they described the Freezing Index (FI), which is the quotient of the power spectral density (PSD) from 3 to 8 Hz (Freezing Band, FB) and the PSD from 0.5 to 3 Hz (Walking Band, WB) [21]. When the FI exceeds an identified threshold (Freezing Threshold, FTH), a FoG episode is considered to have occurred. Due to the presence of false positives (FP) when the patient is at rest, Bächlin et al., in 2009, introduced the Power Index (PI), defined as the addition of WB and FB, which was compared to the Power Threshold (PTH) to establish if there was any relevant quantity of movement when FI was high [21]. PI indicates the amount of movement, therefore, situations where patients were not moving voluntarily were eliminated. In this final algorithm, a FoG episode is then determined to occur if FI>FTH and PI>PTH. The MBFA algorithm is the most advanced FoG detection algorithm due to its low computational cost and good performance [22–24,26,27,31,46–50].

The MBFA algorithm has been widely employed to analyse FoG, although usually in laboratory conditions and very often with low patient numbers. Jovanov et al. implemented this algorithm in real time, although only one patient was used to test the algorithm. Furthermore, no results on sensitivity and specificity were reported [22]. Zabaleta et al. analysed FoG by means of a triaxial accelerometer and a biaxial gyroscope in different locations of the lower limbs. The main features employed were the FI in conjunction with power spectral densities changes. They were capable of correctly detecting 82.7% of FoG episodes with inertial sensors located at each ankle, though only in 2 PD patients [24]. Other attempts to analyse FoG include Stamatakis et al. [51] and Takac et al.[44], but they only used one patient to test the system for PD gait extraction and a system for contextualising FoG events, respectively.

In recent years, Niazmand et al. (2011) presented the Mimed-pants [26], a washable jogging pants with five integrated accelerometers. They employed MBFA for detecting FoG achieving an 88.3% on sensitivity and 85.3% on specificity in 6 PD patients in a short and controlled test focused on inducing FoG regardless of a false positives test. In a recent work in 2012, Zhao et al. [46] performed an embedded algorithm based in the MBFA approach within the Mimed-pants system obtaining 81% for sensitivity with 8 PD patients using similar tests. More recently, Mazilu et al. proposed new online algorithms using 3 accelerometers and comparing different machine learning classifiers that exploit MBFA features, as well as few other ones, in 10 PD patients [48]. Results obtained showed higher than 95% for specificity and sensitivity with different classifiers. However, tests were performed under controlled conditions and, furthermore, the validation methodology overestimated the performance measures since classifiers were trained, iteratively, with all window signals available from a patient except for one window, which was used to obtain the performance values. Therefore, training and testing patterns were very similar, which is very different from normal ADLs. Thus, the reported specificities and sensitivities would be expected to dramatically decrease free-living conditions. In 2013, Moore et al. published their most recent work focused on the MBFA. In this work, they compared different configurations applying the same algorithm on 25 PD patients, among whom 20 had FoG episodes. Different window sizes, sensor locations, and different values for FTH and PTH were evaluated in order to find the optimal conditions. Results showed better performances with longer windows; however, with a long window size, Moore et al. reported a relevant loss of sensitivity at short episodes that, paradoxically, are the most frequent in PD patients [27]. In a more complex test than the one performed previously [20] using up to 7 sensors and a longer test protocol, they achieved sensitivity and specificity over 70% but, in some configurations (window size: 7.5s) with the system worn on the back of the waist, both sensitivities and specificities were over 80%. In a different approach, Tripoliti et al. proved different features and locations in order to also know the best configuration [52]. The work was performed with 5 PD patients and under controlled conditions, with a specific protocol designed to elicit FoG and without FP protocol tests. In this work, they also used 2 gyroscopes along with 6 accelerometers located in different body parts. With all the sensors worn, they obtained an accuracy of 96.11%, 98.74% of specificity and 81.94% of sensitivity with a leave-one-patient-out method. On the other hand, with a single IMU on the waist they report a 75% of sensitivity and 95% of specificity, although the algorithm is not compared with any other method under the same conditions.

In a more recent study from Mazilu’s group, they investigated unsupervised feature learning for building an optimal input vector for a decision tree classifier with the DAPHNET project dataset (10 PD patients). Their approach was compared to an analogous MBFA method in which the FI and the energy of the spectral band between 0.5Hz and 8Hz were evaluated. The training and testing was user-dependent and under controlled conditions. Results enhanced the similar MBFA method by up to 8.1% in terms of F1-score. Another approach was presented by Rodríguez et al. who proposed a method to contextualise FoG episodes by means of an activity recognition algorithm, which rejected those false positives when the patient was sitting and performed activities such as drawing or typing on a laptop. The specificity was improved by up to 5% on average reaching an enhancement of 11.9% in some cases [50]. However, this added-contextualisation method did not contribute to enhanced sensitivity. Other works have studied the variability of gait between a FoG episode and normal gait. Although the results are interesting, they failed to include false positive results and a reliable classification was not performed [53,54]. A recent paper by Zach et al. presented a new methodology to elicit FoG under laboratory conditions which was evaluated with the MBFA algorithm obtaining 75% on sensitivity and 76% on specificity [31].

Finally, Ahlrichs et al., within the REMPARK project [55], used Support Vector Machines (SVM) for detecting FoG episodes in 8 patients with PD at their own homes. The method included tests in different motor states and used a single accelerometer at the waist, achieving accuracies of over 90%. However, specificity was only computed with non-FoG patients, which could lead to unreliable predictions since the models were not tested with PD patients with FoG who move slightly differently to those patients without FoG [30]. Furthermore, the evaluation was performed over a single minute, which is considered too long if cueing is desirable [28].

Tables 1 and 2 summarise the methods presented in the work we have highlighted.

**Table 2:** Summary of published works regarding FoG detection algorithms.

*Work*	*Year*	*Algorithm methods*	*Main significant results presented*
Nieuwboer et al. [39]	2001	Objective measurements and contrast	Reduction of stride length, increase of step cadence compared to normal gait
Han et al.[19]	2003	Statistical test	Observation of frequency response from 6-8Hz in a FoG episode
Hausdorff et al.[38]	2003	Statistical test	Observation of frequency response in FoG from 3-6Hz compared to normal walking
Moore et al. [20]	2008	Threshold based	General threshold: 78.3% success; Personalised threshold: 89.1% success
Daphnet project [56]	2009–2011	Threshold based	General threshold.: 73.1%Sensitivity, 81.6% Specificity; Personalised threshold: 88.6%Sensitivity, 92.8% Specificity
Zabaleta et al. [24]	2008	Linear Classifier through FI variable	82.7% success
Jovanov et al.[22]	2009	Threshold based	Algorithm in real time with fast response. Results of algorithm performance not provided
Maidan et al. [57]	2010	Statistical test	Observation of heart rate evolution during FoG episodes
Knobl et al. [40]	2010	Statistical test	Observation of main differences of a normal gait and gait with FoG
Delval et al.[23]	2010	Threshold based	75–83% Sensitivity, >95%Specificity
Cole et al. [25]	2011	Dynamic Neural Networks	83% sensitivity 97%specificity
Niazmand et al. [26]	2011	Threshold based	88.3% Sensitivity and 85.3% Specificity
Stamatakis et al. [51]	2011	Statistical test	Main differences observation of a normal gait and gait with FoG
Almeida et al. [40]	2012	Statistical test	Gait parameters observation with cues between healthy people and PD patients
Zhao et al. [46]	2012	Threshold based	81.7% Sensitivity, Specificity not provided
Handojoseno et al. [42]	2012	Neural networks through wavelet analysis	75% Accuracy, 75% Sensitivity, 75% Specificity
Mancini et al. [47]	2012	Statistical test	Analysis of frequency ratio and distinguishing objectively PD patients with and without FoG and healthy people
Mazilu et al. [48]	2012	Different classifiers and different temporal-frequency features	Online machine learning system with >95% sensitivity and >95% specificity with some configurations
Takac et al. [58]	2013	Threshold based for the FoG algorithm, Artificial Neural Networks for context algorithms	17 degrees and 0.16 m error (RMSE) for human body orientation
Moore et al.[27]	2013	Threshold based	Contrast of different configurations (window size, sensor location, freezing and power thresholds) Sensitivity and Specificity >70%
Tripoliti et al. [52]	2013	Different classifiers using entropy	96.11% Accuracy
Mazilu et al. [49]	2013	Feature learning with decision tree classifier	Better performance compared to classical methods, approach for detecting pre-FoG
Rodríguez et al. [50]	2014	Threshold based + machine learning context based algorithm	Improvement of 5% in Specificity due to posture contextualisation
Coste et al. [53]	2014	Threshold based through gait parameters	Approach to detect pre-FoG based on gait parameters
Weiss et al. [30]	2015	Statistical test	Observation of gait parameters between freezers and non-freezers
Tay et al. [54]	2015	Threshold based for extracting gait parameters	Observation of variability in gait parameters
Zach et al. [31]	2015	Threshold based	75% Sensitivity, 76% Specificity
Mazilu et al. [43]	2015	Threshold based through Gaussian kernel	Pre-FoG with 71.3% of success
Ahlrichs et al. [28]	2015	RBF kernel function through Support Vector Machines	Accuracies >90%
Maidan et al. [59]	2015	Objective measurements	Observation of variability in levels of haemoglobin just before a FoG episode

## Methods

**Fig 1:**
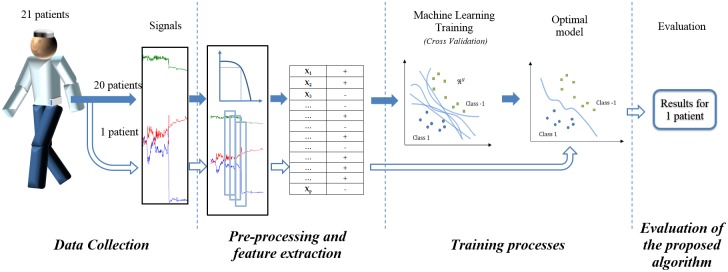
Methods description.

This section describes the new approach proposed by the authors to detect FoG with a supervised machine learning technique and the experimental methods used to evaluate it. The section is divided into 4 sub-sections, (Fig 1) that describes the steps performed in the methodology. At the end of the section, there is a final paragraph devoted to the tests performed to evaluate the MBFA algorithm. The first sub-section describes the database collection procedure, the protocol tests and baseline data from patients. The second sub-section reports the signal processing methods for the SVM model and the extraction of features of the signals based on the online implementation requirements. The third section details the procedure to derive an SVM-based classifier based on a generic model for all patients and in a personalised model. Finally, the evaluation for FoG episodes is described in the fourth section, with respect to the personalised and the generic approaches. In addition, a fifth sub-section has been included, in which the assessment of the MBFA is also explained with the aim to compare the proposed approach to this widely extended algorithm. Although the evaluation of the classifier is the same as the proposed model, the training and feature extraction processes are different; this is detailed in this final section.

### Data collection

The study was approved by local Ethics Commission: Spanish Agency for Drugs and Medical Devices (Spain), Clinical Research Ethics Committee-Galway University Hospitals, Health Department Maccabi Healthcare Services and Ethics Committee Fondazione Santa Lucia. PD patients were diagnosed according the UK Brain Bank [60] criteria and all of them gave their written informed consent to perform the datasets.

A total of 21 patients (3 women and 18 men) with PD participated in the experimental protocol to collect signals. The experimental protocol was part of the REMPARK project [55], which grouped a total of 92 patients with PD (36 women, 56 men) from 4 health centres in Europe, Centro Médico Teknon (Spain), Maccabi Healthcare Services (Israel), Fondazione Santa Lucia (Italy) and National University of Galway (Ireland) [61].

In REMPARK’s experimental protocol, all patients performed the tests both in “OFF” and “ON” states. “OFF” states are those periods where rigidity, bradykinesia or postural alterations appear in PD patients. During the “ON” state, these symptoms are mitigated due to medication effect although other side effects may appear, such as dyskinesias. The inclusion criteria in this clinical protocol were Hoehn & Yahr stage [62] greater than 2 in the OFF state and the absence of dementia according to DSM IV criteria [60]. In this work, which is focused on the detection of FoG episodes, some additional inclusion criteria have been required. First, PD patients should have a FoG Questionnaire (FoG-Q) [15] score above 6, and only those PD patients whose total FoG duration was at least one minute long were selected. Overall, 21 patients out of the 92 patients matched all inclusion criteria for this study (Table 3). The mean age was 69.29±9.72 years old and the mean Hoehn & Yahr stage scoring was 3.07±0.43 in the OFF state. Sixteen patients needed walking assistance and the mean FoG-Q test index was 15.8±4.11, the lowest score being 7 and the highest score 23. Six out of 21 patients did not exhibit dyskinesias, and eleven patients had wearing-off phenomena. At time of the tests, all patients were medicated with optimal doses of levodopa and dopamine agonist therapy. Some baseline data from patient 5 were lost, which is shown as N/A in Table 3; however, they do not affect to the results of this study.

**Table 3:** Clinical characteristics year 2013.

Patient's index	Gender	Age	Year of Diagnosis	Hoehn & Yahr (OFF)	UPDRS part III (OFF)	UPDRS part III (ON)	FoG Questionnaire	MMSE
1	M	83	2008	3	28	14	12	30
2	M	43	2008	2.5	42	5	11	29
3	M	66	2004	4	38	8	17	30
4	M	74	2002	4	53	35	18	25
5	M	72	N/A	3	N/A	N/A	N/A	N/A
6	M	73	2002	3	59	36	17	27
7	M	74	1995	3	53	28	20	26
8	F	69	1997	3	56	15	20	29
9	M	79	1999	3	36	16	12	26
10	M	77	1999	3	43	22	13	25
11	M	72	2002	3	44	29	23	29
12	M	50	2006	2.5	48	9	7	30
13	M	80	2004	3	50	19	23	28
14	M	60	1998	2.5	23	5	17	29
15	F	74	2000	4	33	18	19	27
16	M	60	2001	3	12	3	16	29
17	M	73	2005	3	20	12	16	27
18	M	69	2003	3	23	10	14	29
19	M	74	2008	3	15	11	12	30
20	M	62	2009	3	23	18	13	24
21	F	71	2004	3	27	10	16	26

All patients performed two tests, each approximately 20 minutes long, which comprised a set of scripted activities. All activities were carried out at the patients’ homes, where the probability of eliciting FoG episodes was expected to increase [17]. Medication effect has a strong influence regarding the motor response in PD patients with FoG [11,14,63], thus, tests were performed with and without medication. The first test took place early in the morning before patients had taken their first medication and they felt in OFF state. Thus in the first test, patients completed the different activities without antiparkinsonian medication. Once patients had completed the different activities without medication, they took their usual medication and, at least one hour later, the second test was performed (i.e. ON state). Although the protocol test consisted of specific scripted activities, the test also allowed patients to perform several activities in a free moving manner. Furthermore, the activities were performed in a familiar environment and the patient could proceed with their normal ADLs in a natural way. More specifically, the activities performed in both tests consisted of I) showing the researchers around their home; II) Stand Up and Go test crossing through a doorway and then, turning back (FoG provocation test), which was repeated up to 10 times; III) going outdoors and taking a short walk; and IV) a dual task activity (e.g. reading while carrying an object). In the second test, i.e. following medication, a false positive protocol activity for FoG was additionally performed. This protocol test relies on performing different activities in which the patient executes a short and fast movement repeatedly whose inertial frequency content is similar to a FoG episode. The executed activities were brushing their teeth, painting/drawing/erasing on a sheet of paper and cleaning windows.

A total of 93.03 minutes of FoG episodes were registered (1321 episodes) among these 21 patients, among which 74.12 minutes (79.67%) were registered when patients were in OFF state, 12.35 minutes (13.28%) were registered when patients were in ON state and 6.56 minutes (7.05%) were registered in an intermediate state.

**Fig 2:**
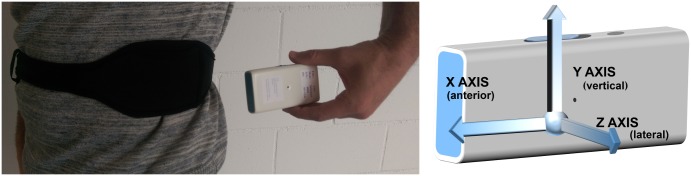
The 9x2 and location of the IMU.

Acceleration signals were captured while patients executed the described protocol with the 9x2 Inertial Measurement Unit (IMU), a small (77x37x21mm^3^) and light (78g with battery) wearable inertial system (Fig 2) which was located at the left side of the waist [64]. This IMU, whose autonomy lasts more than a day, stored the accelerometer data at 200Hz in a microSD card.

All tests were video-recorded and videos were synchronised with the signals recorded by the 9x2 device through a specific method [37]. In order to create a gold standard for the occurrence of FoG episodes, videos were labelled offline by an experienced clinician. Within the MASPARK project [36], all activities, postures and partial FoG episodes were labelled by clinicians.

### Pre-processing and feature extraction

This subsection presents a description of the employed method for pre-processing the signal, which comprises signal conditioning, windowing and feature extraction. Note that, since the proposed algorithm is thought to be implemented in the near future within the 9x2 device for its real-time execution, some computational issues have been taken into account to facilitate this future implementation. For example, windows with reduced amount of samples are considered in order to, first, reduce the latency time of the algorithm; second, to deal with the existing memory restrictions of current microcontrollers, which cannot store inertial information during large periods of time; and third, to prevent the feature extraction process from becoming a heavy computational burden.

The 9x2 provides a 200Hz inertial signal in each of the 3 axis of the accelerometer. This data is resampled at 40Hz since this frequency has been shown to be enough to analyse human movements [65] and it is sufficient to analyse the frequencies obtained during FoG episodes [20]. Once resampled, inertial signals are filtered in order to remove the high frequency noise through a 2^nd^ order Butterworth low-pass filter with a 15Hz cut-off frequency.

The next step is to window the signal. According to Moore and colleagues, the specificity in very small window sizes is very low, thus, it is recommended to work with windows over 2.5 s [27]. In other previous work, Bächlin et al. worked in window sizes of 4s [29], Mazilu et al. showed how FoG detection performance was kept with windows over 3s [48] and Zach et al. showed that window lengths between 2 and 4 s. provided similar results [31]. In order to achieve a similar window length and given that the sampling frequency is 40Hz, a window length of 128 samples (N = 128), i.e. 3.2s of window-length, is proposed. Windows are overlapped at 50% so as to prevent information loss between windows.

**Fig 3:**
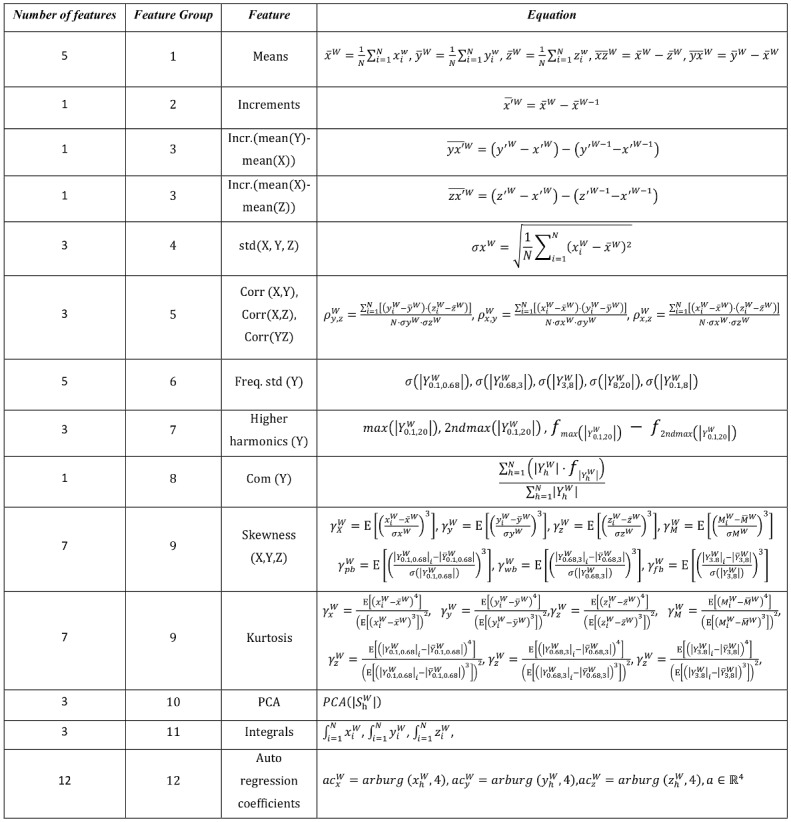
Set of features employed as input vector for the SVM classifier.

With the aim to obtain significant information from each window, some features are extracted. These features will be the input vector, ***x***_*i*_, for the proposed classifier in *Training processes* section, which is based on machine learning techniques. In this work, a total of 55 features are employed, which are presented in Fig 3. Features are briefly described below by grouping them according to their purpose. The number of feature group is noted within brackets.

1. Mean value of each accelerometer axis measurements throughout the window gives the orientation of the inertial system related to gravity in absence of movement.
2. Increments of consecutive windows’ mean values in each accelerometer axis provide the amount of variation performed in each axis of the accelerometer.
3. Difference between the increments of the windows’ mean values among different axes gives the amount of variation among different axes. This feature is significant regarding the detection of postural transitions [66].
4. Standard deviation of each axis indicates the amount of movement performed in a window time.
5. Correlation among each pair of axes gives information about linear relation in different axes, which is useful for detecting normal/continuous gait.
6. Standard deviation on specific spectral bands provides information about the distribution of harmonics in these bands, which define if the harmonics are distributed along the band or if there are significant harmonics. It can give information about stability or specific significant peaks in a band.
7. Highest harmonic peaks are useful for detecting concrete events in specific spectral bands (e.g. high peaks in 0.1Hz to 0.68 Hz band can be considered as a candidate to be a postural transition) [66–68].
8. Spectral density centre of mass gives information about the overall quantity of movement performed and the frequency band in which most of the movement is concentrated.
9. Skewness and kurtosis are employed to characterise the spectral distribution.
10. A change of basis, previously obtained by Principal Component Analysis (PCA), is performed over the energy in the spectral band harmonics from 0.1Hz to 8 Hz. The aim of this is to characterise the harmonics contained in the FB and WB band by features that maximise the variance in this band.
11. Integrals of the accelerometer axis gives information related to the quantity of movement.
12. Auto regression coefficients provide information of how the signal is correlated with a time-shifted version of itself giving the amount of time shift [69].

In order to clarify some feature notation reported in Fig 3, see clarifications below. Let $x_{1}^{W},\ldots \;x_{N}^{W}$ be a set of samples in a certain window *W*. The Short Time Fourier Transform (STFT) is composed of complex values $X_{1}^{W},\ldots \;X_{N}^{W}$ obtained following the next equation:

$$X_{h}^{W}=\sum_{n=1}^{N}x_{n}e^{-i2\pi h\frac{n}{N}}$$ (1)

where *h* = 1, …, *N*. The output of the STFT ($X_{h}^{W}$) is a set of N complex numbers, each one of them representing the amplitude and phase of a harmonic. Let $\left|X_{f_{1\;},f_{2\;}}^{W}\right|=\sum_{h=f1}^{f2}\left|X_{h}^{W}\right|$ be, hence, a set of values representing the sum of the absolute value of $X_{h}^{W}$, where *f*_1_ and *f*_2_ are the upper and lower frequency harmonics denoting the set of harmonics establishing the band to be analysed in the current *W* window. This parameter is employed in the features group 6 to 10. Otherwise, let $M_{i}^{W}=\sqrt{x_{i}^{2}+y_{i}^{2}+z_{i}^{2}}$ be the modulus of the 3 accelerometer axes in a sample *i* and a window *W*. In addition, the highest and the second highest harmonic within the frequency spectrum analysed are denoted as max (*A*) and 2ndmax (A) where $A=\left[A_{1}^{W},\ldots \;A_{N}^{W}\right]$ is the set of harmonics from a signal axis (feature group 7).

Finally, the Principal Component Analysis (PCA) has also been employed, as described below. Let $\left|S_{\text{h}}^{W}\right|=\left|X_{h}^{W}\right|+\left|Y_{\text{h}}^{W}\right|+\left|Z_{\text{h}}^{W}\right|$ be the summation of harmonics for each accelerometer axis, where *h* = 0.1, …,8 *Hz*, so that a vector $S^{W}=[S_{1}^{W},\ldots ,S_{\text{N}}^{W}]$ is obtained, which represents the spectral information in the [0.1, 8] Hz band in a certain window. PCA has been used to reduce the dimensionality of these harmonics. More specifically, PCA has been applied to a set of ***S***^*W*^ vectors organised in a matrix $M={\left[S^{1}{}^{{}^{T}}\;S^{2}{}^{{}^{T}},\ldots ,\;S^{L}{}^{{}^{T}}\right]}^{T}$. These *L* windows are a randomly selected subset of the training data composed by a 10% of the data from each patient, thus containing both FoG episodes and other signals. PCA is applied to in order to obtain the orthonormal change of basis matrix *V* that brings each pattern ***S***^*W*^ from the original space to the latent one. PCA is applied to matrix *M* by first centring the data, and then applying a Singular Value Decomposition (SVD) so that *M* = *U*Σ*V* is obtained. In the SVD process, it was identified that only the first three latent variables contributed with data (i.e. their singular values were significantly higher than the rest). In consequence, only the first three components of the latent representation of ***S***^*W*^, i.e. *V* ***S***^*W*^, are employed as features (feature group 10). Note that the PCA process is only applied once in the training process and a real-time implementation of the feature-extraction process would only require the implementation of the product *V* ***S***^*W*^.

### Training processes

The symptoms related to FoG episodes are user dependent with typical symptoms manifesting as gait abnormalities (e.g. shuffling, leg trembling in place and akinetic episodes) [11]. As each FoG patient experiences these symptoms in different ways, developing an algorithm to detect FoG episodes from inertial signals can be challenging due to the variation between individual signals. To overcome these challenges previous researchers have developed user dependent algorithms capable of detecting FoG episodes with a high degree of accuracy [21,48]. These algorithms require the prior capturing of the patients inertial signals in order to train the algorithm, thus making the algorithm cumbersome to obtain. A less challenging solution would be to develop a generalised algorithm, since it would only require the obtaining of inertial signals from a relatively small cohort of FoG patients in order to train the algorithm for use with any patient. To investigate both types of algorithms and their associated training processes we proposed two methodologies I) a generic model through a leave one out method and II) a model that is personalised to each patient.

#### Generic FoG model selection

In order to obtain a generic (user independent) model, a leave-one-patient-out methodology has been adopted. The model is generic in the sense that it is not personalised to any patient and there is no adjustment made to the model that considers the patient for which the model is tested on. Given the nature of the problem at hand, Support Vector Machines (SVM) have been considered to be the most suitable classifier, due to their high performance method for two-class classification and ease of implementation within microcontrollers [70]. SVM is a machine learning technique that use the *kernel trick* to map input vectors ($x_{i}\in {ℜ}^{N},\;1\leq i\leq p$) to a higher dimensional feature space by *φ*(*x*_*i*_), where typically $\varphi :{ℜ}^{n}\to {ℜ}^{m}$ and subjected by *m* > *n*. The kernel trick enables SVM to avoid bringing *x*_*i*_ to the higher dimensional space by computing the dot product of patterns within ${ℜ}^{n}$.

In our approach, each *x*_*i*_ is represented by 55 parameters (previously described in the *Pre-processing and feature extraction* section) which characterise the current window *W*. The input vectors are associated to one of two different classes, FoG or no-FoG. These classes are represented by the labels *y*_*i*_ = {1, −1}, 1 ≤ *i* ≤ *p*, where *y*_*i*_ = 1 in case of a FoG pattern and *y*_*i*_ = -1 in case of a non-FoG pattern. The set of *y*_*i*_ values is provided by clinical experts with the help of video recordings that were synchronised with their associated inertial signals. In order to maximise the margin, reduce the empirical error and minimise the structural risk, a hyperplane is built to separate the 2 classes [71]. The optimal hyperplane is defined according to the following optimisation problem:

$$minimize\;W\left(\alpha \right)=\sum_{i=1}^{p}\alpha_{i}-\frac{1}{2}\sum_{i,j=1}^{p}\left(\alpha_{i}\alpha_{j}y_{i}y_{j}\cdot K\left(x_{i},x_{j}\right)\right)$$ (2)

$$s.\;t\;0\leq \alpha_{i}\leq C,\;i=1,\ldots ,p$$

in which *C* is the trade-off between the classification margin and misclassification error, and the kernel function *K*(***x***_*i*_, ***x***_*j*_) replaces the dot product *φ*(***x***_*i*_)·*φ*(***x***_*j*_). The kernel used for the FoG classifier is a Gaussian radial basis function (RBF), due to its good performance and generalisation capacity, $K\left(x_{i},x_{j}\right)=e^{-\gamma {\left\|x_{i}-x_{j}\right\|}^{2}}$, where γ is the width of RBF function.

Due to the leave-one-patient-out approach, these parameters must be adjusted with the data from all patients except one. Thus, with this data, a range of values {10^−2^, 10^−1^, …, 10^2^} for *C* and γ are tested through a 10-fold cross-validation method. From the results of the different folds the average sensitivity and specificity is obtained for each combination of values. Optimal parameters for the SVM (*C*_*opt*_, *γ*_*opt*_) are those that maximise the following expression:

$$\left(C_{opt},\;\gamma_{opt}\right)=\text{argmax}\left(\sqrt{Sensitivity_{C,\gamma }\cdot Specificity_{C,\gamma }}\right)$$ (3)

$$s.\;t.\;Sensitivity_{C,\gamma }>70\%,\;Specificity_{C,\gamma }>70\%$$

Once found, the optimal values are used to train the SVM model. The data from the patient that is still unused is then employed to test the model with a specific procedure, which is described in the *Evaluation of the proposed algorithm* section. The entire training process is repeated by keeping apart the data from each patient; in consequence, the number of SVM models obtained is the same as the number of available patients.

#### Personalised FoG model selection

**Fig 4:**
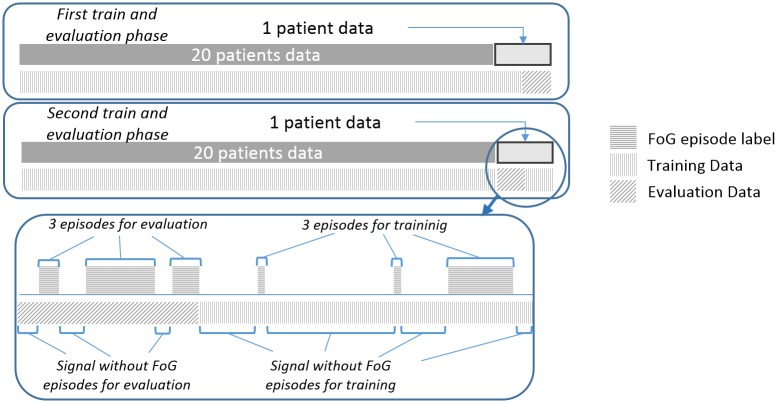
Example of training and evaluation data used in the personalised FoG model selection.

In this section, the proposed user-dependent method is described. In order to obtain a user-dependent model, a modified leave-one-patient-out method has been adopted, which also has been performed by means of SVM. In contrast to the generic model presented in the previous subsection, the personalised model requires labelled inertial signal data from the user in order to obtain the personalised classifier before using it in real life. Therefore, we will use a portion of the data from the patient being tested to train our classifier, and the remaining portion of data will be used to evaluate the algorithm. Signals from the patient being tested are divided into two sets, the first set being composed of 50% of the labelled FoG episodes with the corresponding non-episodic signals, and the second set comprising the remaining FoG episodes and non-episodic windows. Only the first set, i.e. the first half, is included in the training dataset, as shown in Fig 4. We note that the optimal choice of the training data would be to include 50% of the signals into the training dataset (without splitting by episodes) and the remaining 50% into the evaluation dataset. However, there are patients who mostly freeze during the initial data collection while other patients only presented FoG during the second part of the data collection. Therefore, the number of episodes in each dataset would be, in most cases, unbalanced. Hence, the training dataset is chosen to be those parts of the signal from the testing patient that comprise 50% of the FoG episodes, together with the non-episodic signals, as shown in Fig 4. Due to these requirements and the evaluation process, episodes cannot be selected randomly. If FoG episodes were selected randomly, those parts of the signal that do not contain FoG episodes would have to be randomly selected as well. Consequently, the proposed evaluation method would not be valid, since, as shown in the next Section, True Positives (TP) and False Negatives (FN) depend on episode durations, and True Negatives (TN) and False Positives (FP) depend on the amount of detected time spent without a positive detection. Thus, random episode selection would make detection’s temporal line disappear and evaluation by episode would be unfeasible.

Likewise, the same analysis is performed again but swapping the training and evaluation datasets of the testing patient employed to train and to validate the model. In consequence, after first training the model with data from non-testing patients and the first set of the data from the testing patient, a second training takes place. In this case, the second set comprising of the last 50% of the episodes, including non-episodic signals, of the testing patient are included into the training dataset as shown in the lower part of Fig 4. The first 50% of the episodes and its corresponding non-episodic signals are then employed for evaluating the obtained model. Although other percentages of training/evaluation could be tested, in this case, the authors consider that the 50% of FoG episodes’ data contains enough information of non-episodic signals as well.

In this modified leave-one-out approach, data from the non-testing 20 patients represent a clearly greater dataset compared to data from the patient being tested; thus, data from the latter are almost negligible and they would not have any impact in the training process. Hence, it is considered interesting to force the model selection process to learn from the testing patient’s data with more influence than the remaining patients. More specifically, the weight of the testing patient’s data is set to be the same as the data from the other 20 patients. Thus, data belonging to the testing patient, represented by ^*t*^*X* = {***x***_*i*_} is weighted in the following form. Let *n*_*20*_ be the number of patterns of the data belonging to 20 patients used to train the model. On the other hand, let the data from the testing patient be divided into two parts with the first and the second 50% of FoG episodes as previously described, each part containing *n*_f_ and *n*_s_ patterns respectively. The weight given to the those patterns from the training dataset corresponding to the testing patient, when the first half is used, is $w_{t}=\frac{n_{20}}{n_{20}+n_{f}}$. On the other hand, the weight given to those patterns from the training dataset belonging to the 20-patients’ data is $w_{20}=\frac{n_{f}}{n_{20}+n_{f}}$. Accordingly, for the second half, *n*_*s*_ would be used instead of *n*_*f*_. Thus, the weight given to each part corresponds to the prior of the alternative part of the training dataset. These weights are included in the SVM model according to the following equation:

$$maximize\;W\left(\alpha \right)=\sum_{i=1}^{p}\alpha_{i}-\frac{1}{2}\sum_{i,j=1}^{p}\left(\alpha_{i}\alpha_{j}y_{i}y_{j}\cdot K\left(x_{i},x_{j}\right)\right)$$ (4)

s. t.      0≤αi≤C⋅w20,xi∉Xt

0≤αi≤C⋅wt,xi ∈Xt

Finally, the personalised model obtained through the training process will be evaluated over the other set of windows which have not been used in the training according to the episode-based evaluation previously presented. Afterwards, the methodology for obtaining the SVM model will be repeated switching the parts used in the training and evaluation. Thus, the model is prevented from overfitting as only one part of the data is performed by the patient, which may coincide with a specific test and not with ADL. Finally, to ensure each patient’s data is used once as a testing patient the process is completed 21 times.

### Evaluation of the proposed algorithm

To analysis, the performance of each methodology, a rigorous evaluation is performed which follows the methodology previously described [72]. Due to likely situations in which patients are sitting, standing, or lying for long periods of time the proportion of TN (conditions in which a FoG episode was not detected by the algorithm and the patient was not in a FoG episode) becomes extremely large. Similarly, due to situations in which patients experience long FoG episodes the proportion of TP (conditions in which a FoG episode was detected by the algorithm and the patient was in a FoG episode) also becomes extremely large. The purposed evaluation process aims to balance the excessive number of TN and TP that may appear with window-based evaluations of machine learning approaches through the evaluation analysis of episodes instead of the evaluation analysis of windows that contain an episode. Therefore, a FoG episode is evaluated from its beginning until its end as a single episode, instead of being evaluated within every window that contains the FoG episode.

**Fig 5:**
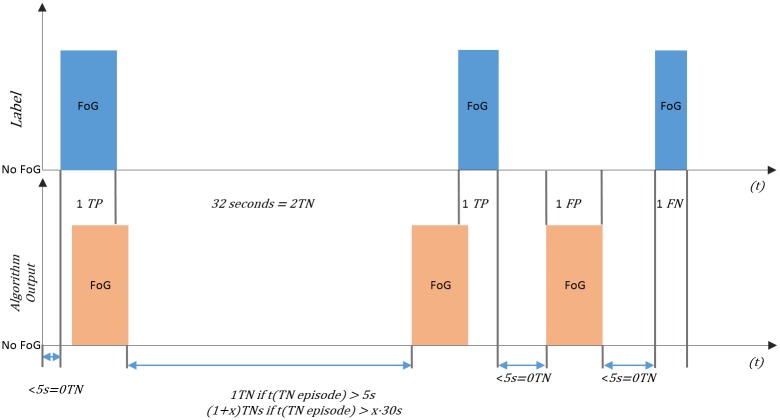
Evaluation method for FoG algorithms.

Given that during the data collection protocol the minimum time taken by patients to perform the false positive test was 38s, it was considered that the maximum time of a single TN episode should be 30 seconds. Thus, for example, if a patient is executing some activity without any FoG episode during 40 seconds, 2 TN are considered. Additionally, a TN episode is only a consideration if the TN episode is longer than 5 seconds as the average duration of a FoG episode in the collected data was 5.14 seconds (Fig 5). Furthermore, TP, FN, and FP are evaluated from Fig 5 as:

- A single TP is considered if a FoG episode was detected by the algorithm and the patient was clinically labelled as having a FoG episode.
- A single FP is considered if a FoG episode was detected by the algorithm and the patient was clinically labelled as not having a FoG episode.
- A single FN is considered if a FoG episode was not detected by the algorithm and the patient was clinically labelled as having a FoG episode.

### MBFA method assessment

To further analyse the performance of each methodology, a comparison to the most widely used technique of FoG detection is made. Given that the MBFA has been shown to provide good performance (>80% in sensitivity and specificity in a non-user dependent algorithm [27]) and, in addition, the MBFA has been widely used by many authors for detecting FoG [22–24,26,27,31,46–50], the authors consider the MBFA as an accurate baseline method for comparison purposes. Similarly, to our proposed approach, a user-independent (leave-one-patient-out) evaluation has been designed for the MBFA method and compared with the proposed user-independent method described in the *Generic FoG Model Selection* section.

The main parameters to be adjusted within the MBFA method are PTH and FTH. Thus, a range of values for each parameter has been tested for each patient, consisting in values [0,0.5, …,4]. After evaluation each pair of PTH and FTH values provide a certain number of TP, TN, FN and FP that are represented in terms of sensitivity $\left(Sensitivity=\frac{TP}{TP+FN}\right)$ and specificity $\left(Specificity=\frac{TN}{TN+FP}\right)$. These values are used to evaluate the MBFA algorithm as described in the *Evaluation of the proposed algorithm* section. Thus, let $Eval_{k}\left(PTH,FTH\right)\;:{ℜ}^{2}\to {ℜ}^{4}$ be a function which returns the number of *TP*, *FP*, *TN*, *FN* of a given signal from patient *k*. From these values, the sensitivity and specificity of the parameters in detecting FoG episodes are obtained based on the previous formulas, which is represented by *Sens* (*Eval*_*k*_ (*PTH*, *FTH*)) and *Spec* (*Eval*_*k*_ (*PTH*, *FTH*)). Given that a leave-one-patient-out method is employed, a summation for all *Eval*_*k*_ (*PTH*, *FTH*) belonging to all non-testing patients is performed following Eq (5):

$$LOO_{t}\left(FTH,PTH\right)=\sum_{k=1,\ldots ,21}^{k\neq t}Eval_{k}\left(FTH,PTH\right)$$ (5)

The optimal values for PTH and FTH are obtained according to Eq (6) and by using the data from the training dataset. These values are then evaluated through the testing dataset, which is the data belonging to the testing patient, represented as *t* patient in the following equation:

$$PTH_{opt},FTH_{opt}=\text{argmax}\left(\sqrt{Sens\;(LOO_{t}\left(PTH,FTH\right))\cdot Spec\;(LOO_{t}\left(PTH,FTH\right)}\right)$$ (6)

$$s.\;t.\;\;Sens\;(LOO_{t}\left(PTH,FTH\right))>70\%\;\;\wedge \;\;Spec\left(LOO_{t}\left(PTH,FTH\right)\right)>70\%$$

Additionally, a personalised optimisation of the MBFA method has been also performed and compared to the corresponding methodology presented in the *Personalised FoG model selection* section. The user dependent model has also been obtained by including 50% of the episodes from the patient being tested and, in addition, the corresponding windows without episodes (as shown in Fig 5) within the optimisation of PTH and FTH.

Similar to the SVM-based approach presented in the *Personalised FoG model selection* section, the training dataset, which belongs to the patient being tested, is weighted in order to adjust the parameters to the patient’s FoG episodes. Hence, let *Eval*_*t*1_ (*PTH*, *FTH*) and *Eval*_*t*2_ (*PTH*, *FTH*) be the functions which return the number of TP, FP, TN, and FN from, on the one hand, the first half of the signal containing the first 50% of FoG episodes in the *t* patient and, on the other hand, the second half of the signal containing the last 50% of FoG episodes in the *t* patient. The evaluation of the remaining part of the signal from the *t* patient provides the personalised model performance based on TP, FP, TN and FN, which is represented by *PM*_*t*_ (*FTH*, *PTH*). The corresponding specificity and sensitivity are obtained according to:

$$Sens(PM_{t1}\left(FTH,PTH\right))=Sens\;(LOO_{t}\left(PTH,FTH\right))\cdot w_{t}+Sens(Eval_{t1}\left(FTH,PTH\right))\cdot w_{20}$$ (7)

$$Spec(PM_{t1}\left(FTH,PTH\right))=Spec\;(LOO_{t}\left(PTH,FTH\right))\cdot w_{t}+Spec(Eval_{t1}\left(FTH,PTH\right))\cdot w_{20}$$ (8)

Finally, optimal values of *PTH* and *FTH* for patient *t* in the personalised model are obtained by:

$$PTH_{opt},FTH_{opt}=\text{argmax}\left(\sqrt{Sens\;(PM_{tb}\left(PTH,FTH\right))\cdot Spec\;(PM_{tb}\left(PTH,FTH\right)}\right)$$ (9)

s. t.  Sens (PMtb)>70% ^ Spec(PMtb)>70%

The same methodology is then applied for *t*2 in order to evaluate the remaining half of the signals.

## Results and discussion

In this section, the results obtained for the methodologies described in the *Methods* section are reported. To put the results into perspective, the SVM and the MBFA results are shown together. Both SVM and the MBFA methods of FoG detection have been evaluated on a generic model and a personalised model basis. Table 4 presents the specificity, sensitivity and their geometric mean averaged among the 21 PD patients for both methods and both models. The values reported in Table 4 have been obtained by summing the TP, TN, FP and FN values and solving for sensitivity and specificity as reported in the previous sections.

**Table 4:** Results for the generic and the personalised models for the proposed SVM-based approach and the MBFA method. Average specificity, sensitivity and geometric mean, computed as $\sqrt{\text{Specificity}\cdot \text{Sensitivity}}$, for the 21 PD patients are presented.

	Generic model			Personalised model		
	Sensitivity	Specificity	Geometric mean	Sensitivity	Specificity	Geometric mean
SVM	74.7%	79.0%	76.8%	88.1%	80.1%	84.0%
MBFA	81.6%	52.6%	65.6%	89.1%	61.5%	74.0%

Table 4 clearly shows that the personalised models for both the SVM and the MBFA methods outperform the generic models, providing an increase of 7.2% for SVM and 8.4% for MBFA in terms of geometric mean Thus, the detection of FoG episodes are significantly improved when data from the patient being tested is used to train the model or to adjust the MBFA parameters. The resulting increase of 8.4% obtained for the MBFA is consistent with the results reported in [21]. Similarly, the SVM method shows better results by considering the data from the patient being tested, which is an interesting finding that has not been previously reported and that can help to obtain enhanced models for FoG detection.

Regarding the performance of the MBFA and the SVM method, it can be seen that the SVM methods geometric mean is significantly greater than the MBFA methods geometric mean. Specifically, the proposed method outperforms the MBFA method with an increase of 11.2% for the generic model and an increase of 10% for the personalised model. Thus, the detection of FoG episodes are significantly improved using the SVM method in comparison to the MBFA method. In fact, the machine learning approach better generalises the problem in all the results obtained. This is due to the inclusion of several features that characterise in a more accurate way not only the FoG episodes but also those parts with possible FP, thus providing a much higher specificity. In this sense, it is notable that the specificity of the MBFA generic model is 52.6%, while the corresponding SVM-based specificity is 79%.

Table 5 presents the individual patient results obtained by the SVM method for both the generic and the personalised models. These results aim to show how the personalisation model improves the detection of FoG episodes in comparison to the generic model. For example, the geometric mean for patient 1 is only 37.35% for the generic model and 86.2% for the personalisation model. In total, for 16 patients the personalisation provides an increase in the geometric mean which corresponds to a greater detection of FoG episodes. However, in the remaining 5 patients (number 4, 10, 11, 12 and 18), the geometric mean is slightly lower, below 3%. This may occur when the scenario in the first half of the test is different to the second part and, thus, episodes may appear differently. Most patients freeze similarly during the whole test but, however, there are patients who freeze in a different manner inside their homes than outside. Trained models can learn that, in the main, episodes occur in a stereotypical way. When the evaluation is performed outdoors or in a different scenario in the other half of the test the classifier might not work properly and thus the mean could decrease a little compared to the generic SVM.

**Table 5:** Results of the proposed SVM-based approach for both generic and personalised models.

Index	Generic model			Personalised model		
	Sensitivity	Specificity	Geometric mean	Sensitivity	Specificity	Geometric mean
Patient 1	18.89%	73.85%	37.35%	92.08%	80.70%	86.20%
Patient 2	46.77%	51.88%	49.26%	80.95%	59.81%	69.58%
Patient 3	39.58%	92.21%	60.41%	100.00%	90.91%	95.35%
Patient 4	100.00%	60.00%	77.46%	96.55%	57.89%	74.77%
Patient 5	92.59%	83.05%	87.69%	98.28%	89.29%	93.67%
Patient 6	100.00%	68.99%	83.06%	97.50%	83.04%	89.98%
Patient 7	86.96%	86.36%	86.66%	91.67%	83.33%	87.40%
Patient 8	100.00%	57.01%	75.50%	96.72%	73.91%	84.55%
Patient 9	83.33%	53.73%	66.91%	80.00%	75.00%	77.46%
Patient 10	93.59%	73.49%	82.94%	84.88%	77.01%	80.85%
Patient 11	93.31%	92.59%	92.95%	94.67%	88.10%	91.32%
Patient 12	83.95%	60.98%	71.55%	77.91%	61.04%	68.96%
Patient 13	91.25%	66.41%	77.84%	86.42%	77.34%	81.76%
Patient 14	88.14%	64.91%	75.64%	81.97%	74.77%	78.29%
Patient 15	98.73%	77.78%	87.63%	96.20%	82.35%	89.01%
Patient 16	92.73%	62.34%	76.03%	91.07%	71.43%	80.65%
Patient 17	70.31%	91.67%	80.28%	78.79%	91.49%	84.90%
Patient 18	100.00%	88.89%	94.28%	92.31%	92.21%	92.26%
Patient 19	82.35%	93.85%	87.91%	92.31%	96.88%	94.56%
Patient 20	32.35%	75.47%	49.41%	51.43%	85.71%	66.39%
Patient 21	64.71%	92.68%	77.44%	88.24%	89.74%	88.99%
**Average**	**79.03%**	**74.67**%	**75.15%**	**88.09**%	**80.09**%	**83.66**%

Some previous papers have reported results of sensitivity and specificity over 90%, which are higher than those shown in this paper. However, some issues are evident in those works [28,48,52].

The evaluation method consists of a leave one window out, which actually trains the model with all the windows (including the testing patient) except for one. Therefore, the testing signals are very similar to the trained signals, resulting in very high accuracies. In addition, the leaving one window out approach considers each window either as a positive or a negative case, which overestimates specificity and sensitivity values, as presented in the previous sections. In the majority of the papers, results have been obtained by considering either laboratory conditions or the use of multiple sensors, which increases the accuracy of FoG detection.

In this work, a more reliable evaluation method has been employed, which consists in evaluating through the leave one patient out method. Using this method, at least half of the signals from the testing patient (for the personalised model) has been omitted in order to properly test the model. Additionally, our results have been obtained in the patients’ own home and by using a single sensor located on the waist. The waist location has been shown to be comfortable for users [32] but, in contrast, the detection of FoG accuracy is not as accurate as that obtained by setting the inertial systems on the feet [21]. In consequence, the additional difficulty provided through these conditions also explain the lower results obtained.

With a specificity of 61% and sensitivity values of approximately 50% on average, it is evident that the evaluation of MBFA is reduced due to the new evaluation methodology. In addition, the effect of performing the dataset in patient’s home environments and while performing some false positive tests, suggests the MBFA may not be very reliable in a home environment.

The proposed evaluation method assesses the algorithm for FoG detection in relation to the number of episodes, by balancing the number of TN, TP, FN and FP. However, this method is not able to determine the length of each episode. Given that the processing method is performed through windows, the resolution of the episode can only be 1.6 seconds and the minimum FoG episode time is 3.2 seconds according to the specifications reported in the *Pre-Processing and feature extraction* section.

So far, the MBFA model has been seen as an optimal model due to its low computational cost and good performance. Compared to our model, the computational burden is significantly lower; nonetheless, the accuracies obtained are also lower. However, with recent technological advances, the inclusion of virtual digital signal processors within the microcontrollers and the relevant growth of performance with low power consumption makes it feasible to implement real-time machine learning classifiers with several support vectors and wide feature vectors. In spite of this, the authors considered that the models obtained by the proposed approach should be optimised in the future before being implemented in real time. The model purposed has been optimised by maximising the product of sensitivity and specificity but not including any constraint to minimise support vectors and, in addition, it has no attempt to select features among the 55 proposed features undertaken. Regarding the online optimisation, the non-user dependent model presented employs an average of 6790±387 support vectors among the different leave-one-out models. Considering 4 bytes for each floating point number, an average of 1494KB of memory is required in order to store the SVM model. On the other hand, the user dependent model uses 9221±520 support vectors requiring 2028KB. These models may require a considerable portion of the memory available on some low to moderate power consumption and high-performance microcontroller such as Cortex-M4 ARM microcontrollers. Thus, a reduction of support vectors may be done, for instance, by means of Separable Case Approximation method [73]. Using this method, the non-user dependent model is reduced from 7147 support vectors to 4598, reducing the amount of memory needed by 30%. Although the proposed algorithm has been developed to be implemented online within a microcontroller, and there are low power high-performance microcontrollers that could support the required 1011KB (corresponding memory for 4598 support vectors), a reduction of features should be done, together with further removal of support vectors, in order to minimise the computational burden on the prediction phase. However, this may reduce performance of the algorithm, thus, the trade-off between performance and computational burden, which directly affects the microcontroller capacity and increases its consumption has to be considered.

## Conclusions

The MBFA is a method to detect FoG episodes with the analysis of frequency features obtained by an accelerometer signal of a wearable system. This method has been used in numerous studies [22–24,26,27,31,46–50] due to its simplicity (just the optimisation of 2 features) and its high accuracy in detecting FoG episodes (>80%, according to Moore et al. [27]). However, the method has only been tested under laboratory conditions without taking into account situations which could lead to false positives. As far as the authors know, the evaluation method does not consider those situations where long periods of negative outputs such as sitting, lying, or standing, overstate the specificity results.

This paper presents a supervised machine learning approach to detect FoG that has been trained and evaluated with an inertial dataset obtained from an IMU located at patients’ waist while the PD patients were at home [37]. Two methodologies have been proposed and evaluated based on the leave one out method. On the one hand, a generic model that is not adjusted to any patient has been proposed, and, on the other hand, a personalised model, which is trained with part of the inertial signal from the patient undergoing testing. Both generic and personalised model selection approaches have been assessed with the MBFA and the proposed machine learning method. Furthermore, an optimisation method to select the MBFA model has been employed. The evaluation has been performed by balancing the number of true positives and true negatives and also evaluating episode instead of the window, leading to a more reliable assessment. For the personalised model, only the 50% of the FoG episodes including those non-episodic signals have been used for training, enabling the rest of the signals to be used for evaluation. This percentage has been selected given that some patients have a low number of FoG episodes, this way, the evaluation set is larger. Furthermore, validation of results is more significant if evaluation percentage is larger since the classifier avoids overfitting, reinforcing, thus, the performance of the model.

Our results show how the personalised models outperform the generic models by on average by 10%. However, and as stated in the *Personalised FoG model selection* section, it requires a data capture and data training process, which is not necessary with the non-user dependent model. This process involves taking data from patients with video recording to capture different FoG episodes at the patients’ home with a specific protocol test to elicit FoG episodes. This method of data collection can be cumbersome to obtain for the patient.

On the other hand, the machine learning approach enhances significantly the results obtained with the MBFA model. Evaluations per episode show that the machine learning approach either increases both sensitivity and specificity or may only increase one, leave the other unchanged. The geometric mean of both sensitivity and specificity and their average have been used to compare both models (MBFA and SVM). SVM enhances these values by 8% and 11%, respectively. Improvements might be explained by 2 factors. Firstly, the capacity of generalisation of a machine learning method in comparison to the 2 thresholds based MBFA method. Secondly, is the inclusion of several features, which better characterise the episodes and the non-episodic movements. Since the database was constructed from data collected in real environments, situations that are more reflective of real conditions could be used in order to strengthen the classifier against false positives, achieving much better specificities. However, the algorithm needs some optimisation in order to be implemented in a microcontroller of a wearable device for real-time applications. The most relevant issue is the number of support vectors, which should be reduced as shown in the *Evaluation of the proposed algorithm* section. This reduction would lower the computational burden.

Results obtained provide a sensitivity and specificity geometric mean of 84% in the personalised model and of 76% in the generic model, which are significant enough to evaluate FoG at patients’ home. These results have been obtained under challenging conditions, at patients’ home and with a rigorous evaluation process in order to balance the number of true positives and true negatives.

As far as authors know, this algorithm is the first to achieve high sensitivities and specificities considering an online implementation using a balanced assessment per episode through patients’ home training, which included a false positive test. This algorithm opens up the possibility to rigorously assess FoG in PD patients in real time with the aim of acting quickly with some cueing modalities [8,74]. Additionally, given that medication considerably affects FoG frequency and severity in some patients [63], this algorithm could help to accurately tailor medication regimes in future.
